# Supplementary material for: A synaptic wiring diagram underlying modality-specific ascending pathways of the superficial spinal dorsal horn
Source: bioRxiv. 2026 Oct 1:2026.09.27.754843. Preprint. [Version 1] doi: 10.64898/2026.09.27.754843 (PMC13648488; doi:10.64898/2026.09.27.754843)
Supplement: Supplementary file 1 [file NIHPP2026.09.27.754843v1-supplement-1.pdf]

bioRxiv preprint doi: https://doi.org/10.64898/2026.09.27.754843; this version posted October 1, 2026. The copyright holder for this preprint (which was not certified by peer review) is the author/funder, who has granted bioRxiv a license to display the preprint in perpetuity. It is made available under a CC-BY 4.0 International license.

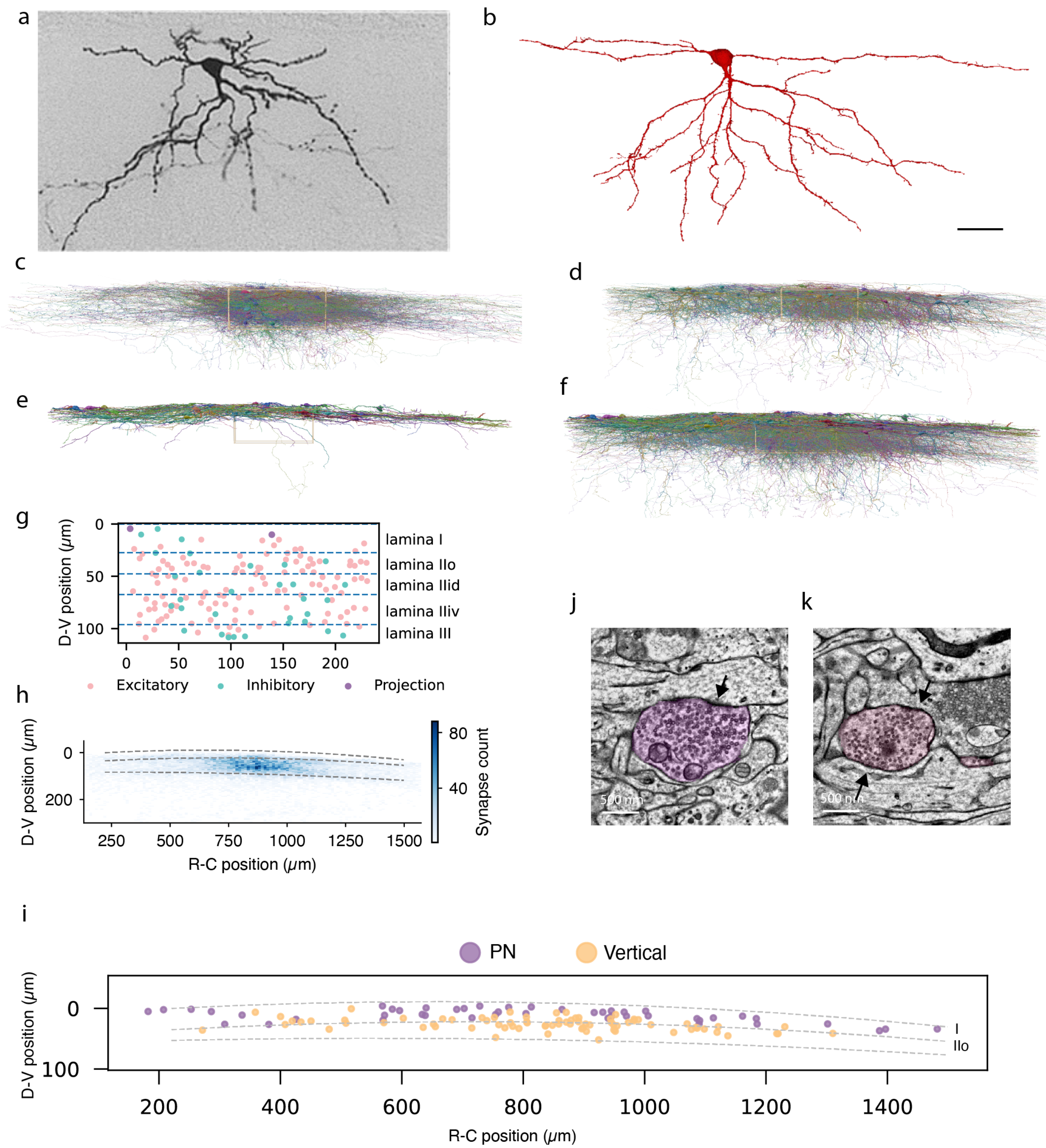


**Figure S1 | Dense reconstruction of ExNs**

**a**, Classic vertical cell labeled by biocytin filling. Figure adapted from (Grudt and Perl, 2002).

**b**, A vertical cell identified in the EM volume. Vertical cells can be reliably recognized by their characteristic laminar location and ventrally directed dendrites. Scale bar, 30 μm.

**c**, Dense reconstruction of excitatory neurons (*n* = 100) within the ROI indicated by the yellow

bioRxiv preprint doi: https://doi.org/10.64898/2026.09.27.754843; this version posted October 1, 2026. The copyright holder for this preprint (which was not certified by peer review) is the author/funder, who has granted bioRxiv a license to display the preprint in perpetuity. It is made available under a CC-BY 4.0 International license.

box, which spans 230 × 100 × 31 μm along the rostrocaudal, dorsoventral, and mediolateral axes, respectively.

**d**, Reconstructed vertical cells (n = 77) shown relative to the ROI.

**e**, Reconstructed lamina I projection neurons (n = 46) shown relative to the ROI.

**f**, All reconstructed neurons included in this study (n = 223) shown relative to the ROI.

**g**, Soma distribution of ExNs, IhNs, and PNs within the reconstruction ROI.

**h,** Spatial distribution of output synapses from reconstructed ExNs in the dSC1 volume. Color intensity represents synapse density. Dashed lines indicate the boundaries between white matter and lamina I, lamina I and lamina II, and lamina II and lamina III. Only the superficial 300 μm region below the white matter–lamina I boundary is shown.

**i**, Soma distribution of all reconstructed vertical cells and PNs across the EM volume. These cell types were reconstructed throughout the volume to better capture sensory processing circuitry.

**j**, Example excitatory synapse. Excitatory neurons in laminae I-II form classic asymmetric synapses with a prominent postsynaptic density (arrow).

**k**, Example inhibitory synapse. Inhibitory neurons in laminae I-II form classic symmetric synapses with little or no postsynaptic density (arrow). This neuron also forms an axoaxonic synapse onto a sensory terminal, further supporting its inhibitory identity.

bioRxiv preprint doi: https://doi.org/10.64898/2026.09.27.754843; this version posted October 1, 2026. The copyright holder for this preprint (which was not certified by peer review) is the author/funder, who has granted bioRxiv a license to display the preprint in perpetuity. It is made available under a CC-BY 4.0 International license.

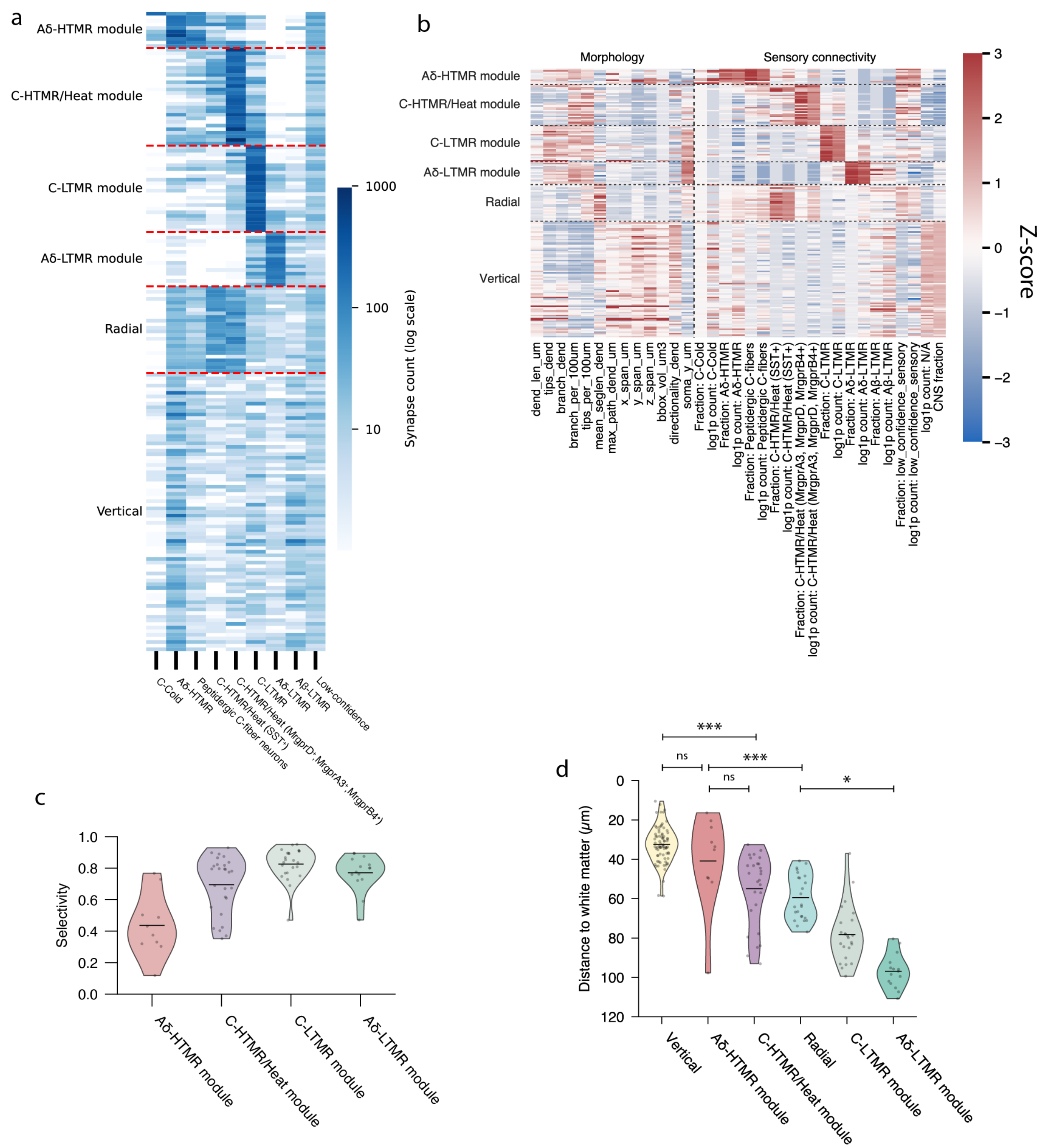


**Figure S2 | ExN clustering**

**a**, Raw sensory neuron synapse counts onto individual ExNs. Rows show single ExNs sorted by subtype; columns indicate sensory neuron subtypes; color denotes synapse count.

bioRxiv preprint doi: https://doi.org/10.64898/2026.09.27.754843; this version posted October 1, 2026. The copyright holder for this preprint (which was not certified by peer review) is the author/funder, who has granted bioRxiv a license to display the preprint in perpetuity. It is made available under a CC-BY 4.0 International license.

**b**, Z-score heatmap of the morphological and synaptic input features used for UMAP embedding. ExNs within the same subtype show similar feature profiles; features are described in Methods.
**c**, Selectivity of sensory input across ExN modules. Violin plots show, for each sensory-defined ExN module, the fraction of total sensory synaptic input contributed by the module's defining sensory subtype (dots, individual ExNs; line, median).
**d**, Depth of ExN somata measured as distance from the white matter boundary.
*P<0.05;**P<0.01;***P<0.001, using a Kruskal–Wallis test, and Dunn posthoc test with Holm–Bonferroni-correction. Vertical cells, n=77; radial cells, n=24; Aδ-LTMR module ExNs, n=15; C-LTMR module ExNs, n=24; Aδ-HTMR module ExNs, n=10; C-HTMR/Heat module ExNs, n=27.)

bioRxiv preprint doi: https://doi.org/10.64898/2026.09.27.754843; this version posted October 1, 2026. The copyright holder for this preprint (which was not certified by peer review) is the author/funder, who has granted bioRxiv a license to display the preprint in perpetuity. It is made available under a CC-BY 4.0 International license.

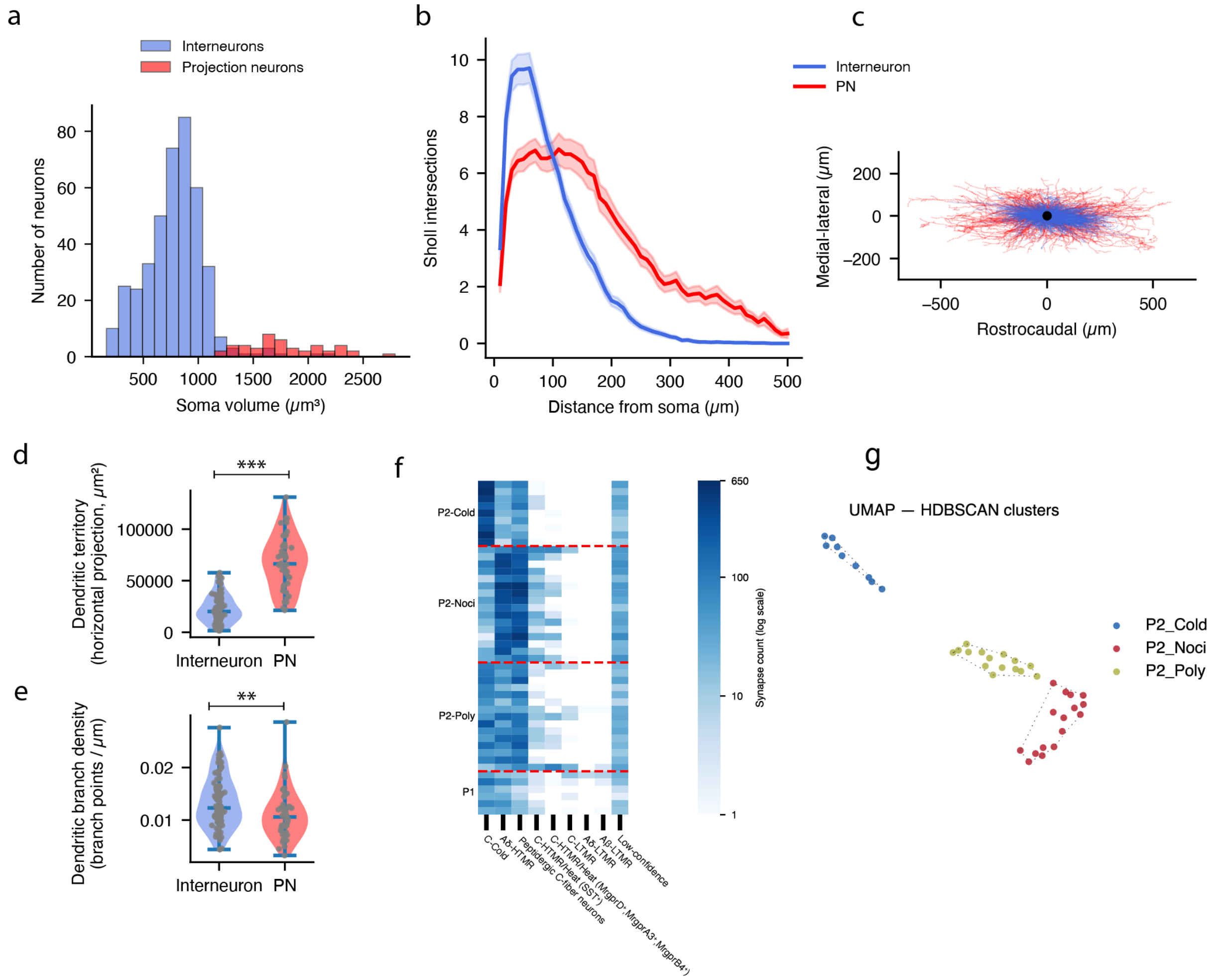


**Figure S3 | PN morphologies and clustering**

**a**, Soma volume distribution of lamina I neurons. Putative projection neurons (n = 45) have a mean soma size 1800.9 ± 365.5 μm³ (mean ± SD), lamina I interneurons (n = 411) have mean soma size 787.3 ± 269.0 μm³ (mean ± SD). One additional PN, located at the edge of the EM

bioRxiv preprint doi: https://doi.org/10.64898/2026.09.27.754843; this version posted October 1, 2026. The copyright holder for this preprint (which was not certified by peer review) is the author/funder, who has granted bioRxiv a license to display the preprint in perpetuity. It is made available under a CC-BY 4.0 International license.

volume, had a partially reconstructed soma and was therefore excluded from soma-volume quantification; inspection of its cross-sections nevertheless confirmed its large soma size.

**b**, Sholl analysis of the dendritic arborizations of 46 lamina I PNs and 85 interneurons. PNs displayed complex dendritic branching extending over a larger spatial range. For **Figures S3b-e**, we included in the analyses 85 well-reconstructed interneurons where their dendritic and axonal compartments could be automatically split.

**c,** Dendritic fields aligned to the soma. Individual dendritic arbors from PNs (red, n = 46) and interneurons (royal blue, n = 85) were centered on the soma position to compare their spatial organization. PNs displayed broader rostrocaudal dendritic territories, whereas interneurons showed more compact dendritic fields.

**d,** Quantification of dendritic territory in the horizontal view as **c**. PNs exhibited significantly larger dendritic fields than lamina I interneurons, measured by the area of the convex hull encompassing dendritic processes in the horizontal projection (PN: mean 67,030.8 ± 26,362.6 µm²; interneurons: mean = 22,724.8 ± 13,471.0 µm²; $p = 2.2 \times 10^{-16}$, $n_{PNs} = 46$, $n_{interneurons} = 85$, Mann–Whitney U test).

**e**, Quantification of dendritic branching density. Interneurons exhibited slightly higher dendritic branch density compared with PNs. Branch density was calculated as the number of branch points divided by total dendritic length (PN: mean 0.011± 0.005 branch points per µm vs. interneuron: mean 0.013 ± 0.005; $p = 0.0076$, $n_{PNs} = 46$, $n_{interneurons} = 85$, Mann–Whitney U test).

**f**, Raw sensory synapse counts onto individual PNs. Rows show single PNs sorted by subtype. Columns indicate sensory afferent subtypes; color denotes synapse count.

**g**, UMAP embedding of PNs using synaptic input features. Dashed outlines indicate HDBSCAN-identified clusters; colors denote the final subtype assignments.

bioRxiv preprint doi: https://doi.org/10.64898/2026.09.27.754843; this version posted October 1, 2026. The copyright holder for this preprint (which was not certified by peer review) is the author/funder, who has granted bioRxiv a license to display the preprint in perpetuity. It is made available under a CC-BY 4.0 International license.

a

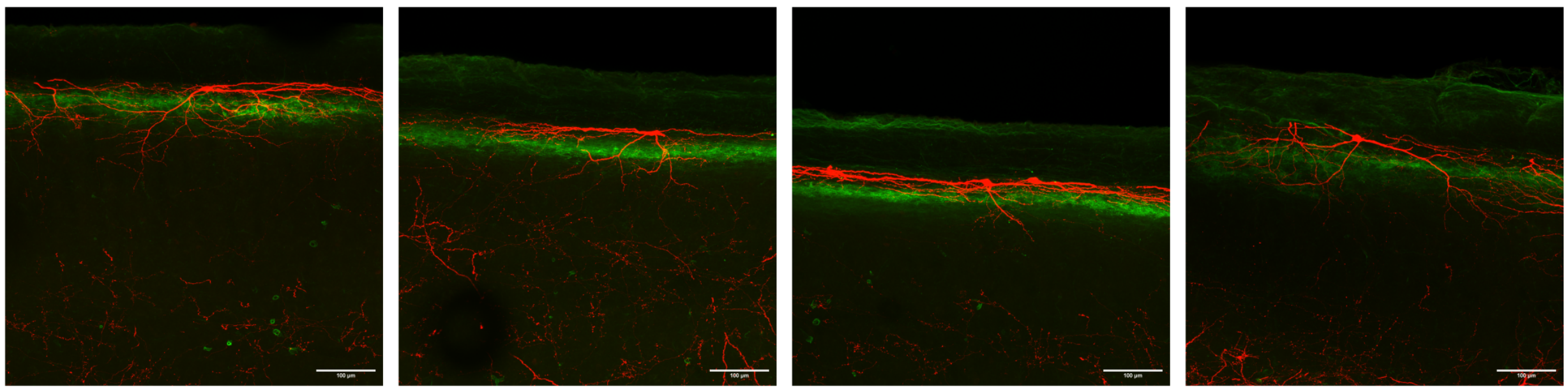

*Gpr83*$^{CreER}$; *Phox2a*$^{Flp}$; *Ai65*

b

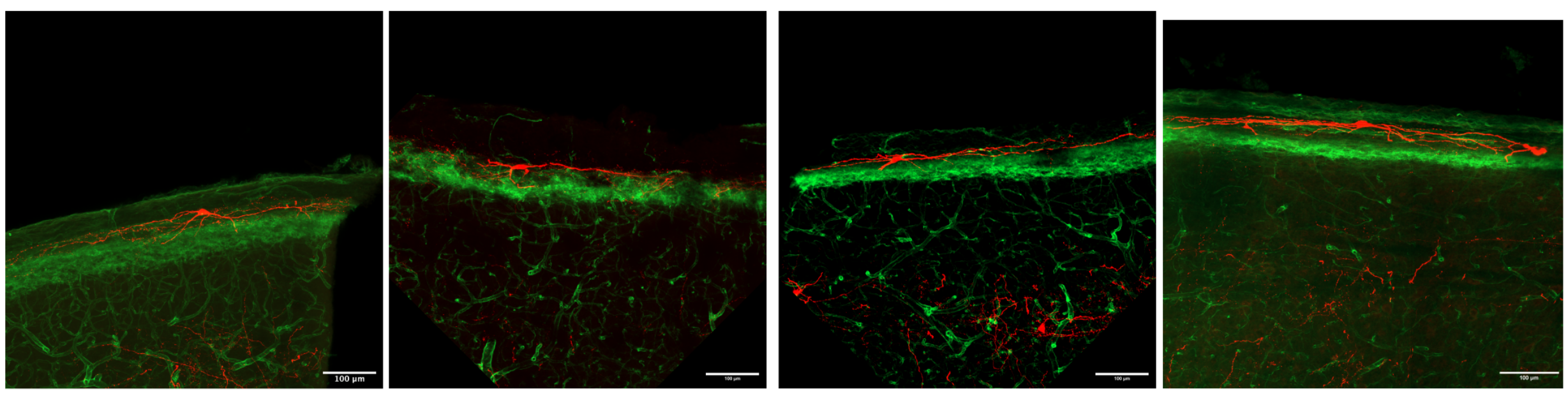

*Tacr1*$^{CreER}$; *Phox2a*$^{Flp}$; *Ai65*

**Figure S4 | Sparse labeling of *Gpr83*⁺ and *Tacr1*⁺ lamina I projection neurons**

**a**, Confocal microscopy of *Gpr83*$^+$ lamina I PN morphology. *Gpr83*$^+$ projection neurons (red) extend dendrites ventrally, below the lamina IIid boundary marked by IB4 staining (green).

bioRxiv preprint doi: https://doi.org/10.64898/2026.09.27.754843; this version posted October 1, 2026. The copyright holder for this preprint (which was not certified by peer review) is the author/funder, who has granted bioRxiv a license to display the preprint in perpetuity. It is made available under a CC-BY 4.0 International license.

**b**, Confocal microscopy of *Tacr1*$^+$ lamina I PN morphology. *Tacr1*$^+$ projection neurons (red) extend dendrites that are largely confined to lamina I and dorsal lamina IIo, remaining above the lamina IIid boundary marked by IB4 staining (green). Scale bar, 100 μm (a,b).

bioRxiv preprint doi: https://doi.org/10.64898/2026.09.27.754843; this version posted October 1, 2026. The copyright holder for this preprint (which was not certified by peer review) is the author/funder, who has granted bioRxiv a license to display the preprint in perpetuity. It is made available under a CC-BY 4.0 International license.

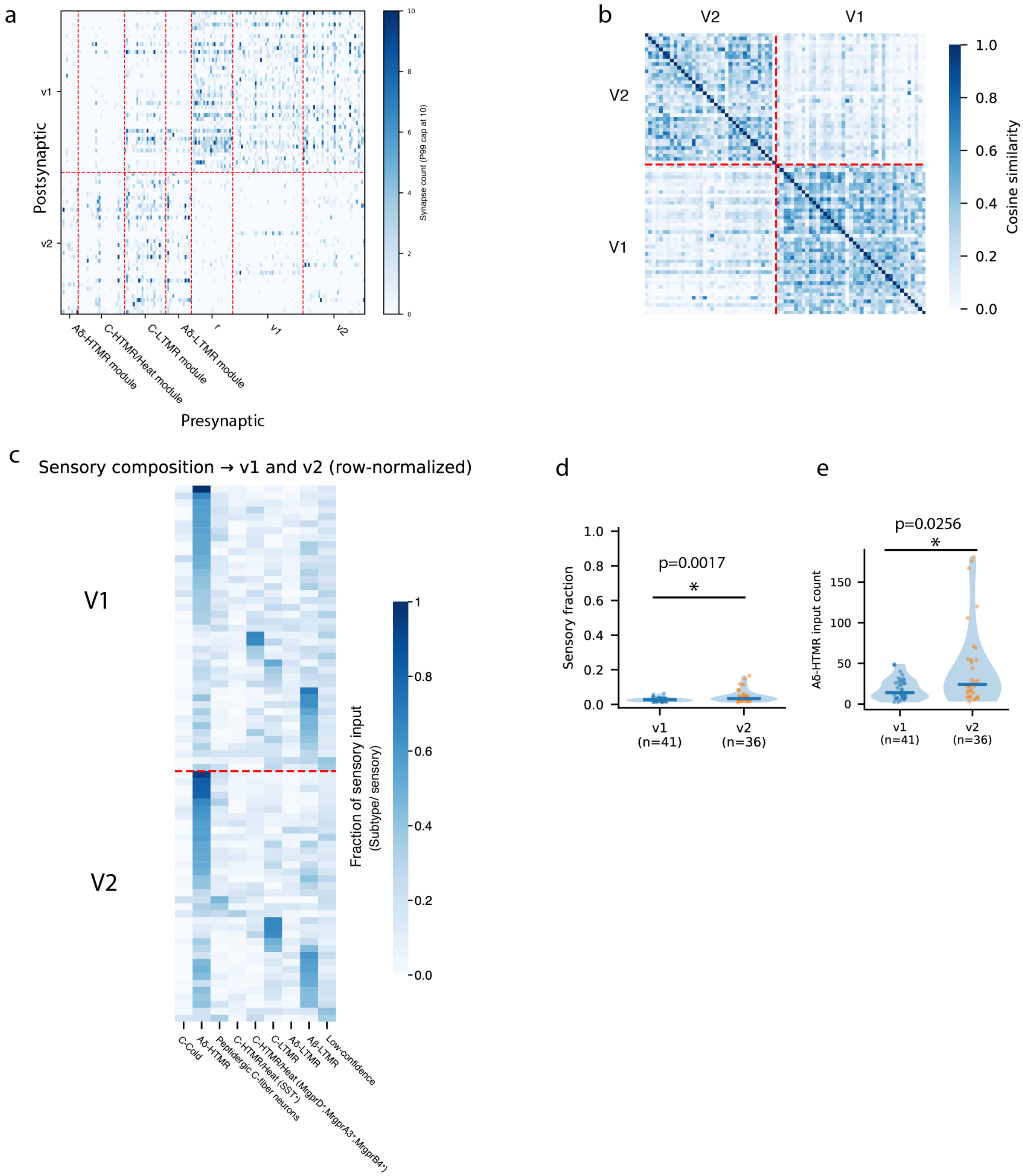


**Figure S5 | Vert1 neuron and Vert2 neuron connectivity**

**a**, ExN inputs onto the two vertical cell subpopulations. Vert1 neurons receive input from the C-LTMR module, radial cells, Vert2 cells, and recurrent input from other Vert1 neurons, whereas Vert2 neurons primarily receive input from the sensory modules.

**b**, Cosine similarity matrix of vertical-cell ExN input profiles. Vertical cells segregate into two

bioRxiv preprint doi: https://doi.org/10.64898/2026.09.27.754843; this version posted October 1, 2026. The copyright holder for this preprint (which was not certified by peer review) is the author/funder, who has granted bioRxiv a license to display the preprint in perpetuity. It is made available under a CC-BY 4.0 International license.

populations (Vert1 and Vert2) defined by spectral clustering of ExN input features.

**c**, Fraction of sensory inputs from each afferent subtype onto Vert1 and Vert2.

**d**, Fraction of sensory neuron versus non-sensory neuron synaptic input onto Vert1 and Vert2 neurons. Vert2 neurons receive slightly, but significantly more direct sensory input ($p = 0.0017$, Mann–Whitney test, v1:n=41, v2: n=36). Note that both Vert1 and Vert2 neurons receive very few direct sensory neuron synaptic inputs.

**e**, Raw Aδ-HTMR synapse counts onto Vert1 and Vert2. Vert2 neurons receive slightly, but significantly more Aδ-HTMR inputs compared to Vert1 neurons ($p = 0.0256$, Mann–Whitney test, v1:n=41, v2: n=36).

bioRxiv preprint doi: https://doi.org/10.64898/2026.09.27.754843; this version posted October 1, 2026. The copyright holder for this preprint (which was not certified by peer review) is the author/funder, who has granted bioRxiv a license to display the preprint in perpetuity. It is made available under a CC-BY 4.0 International license.

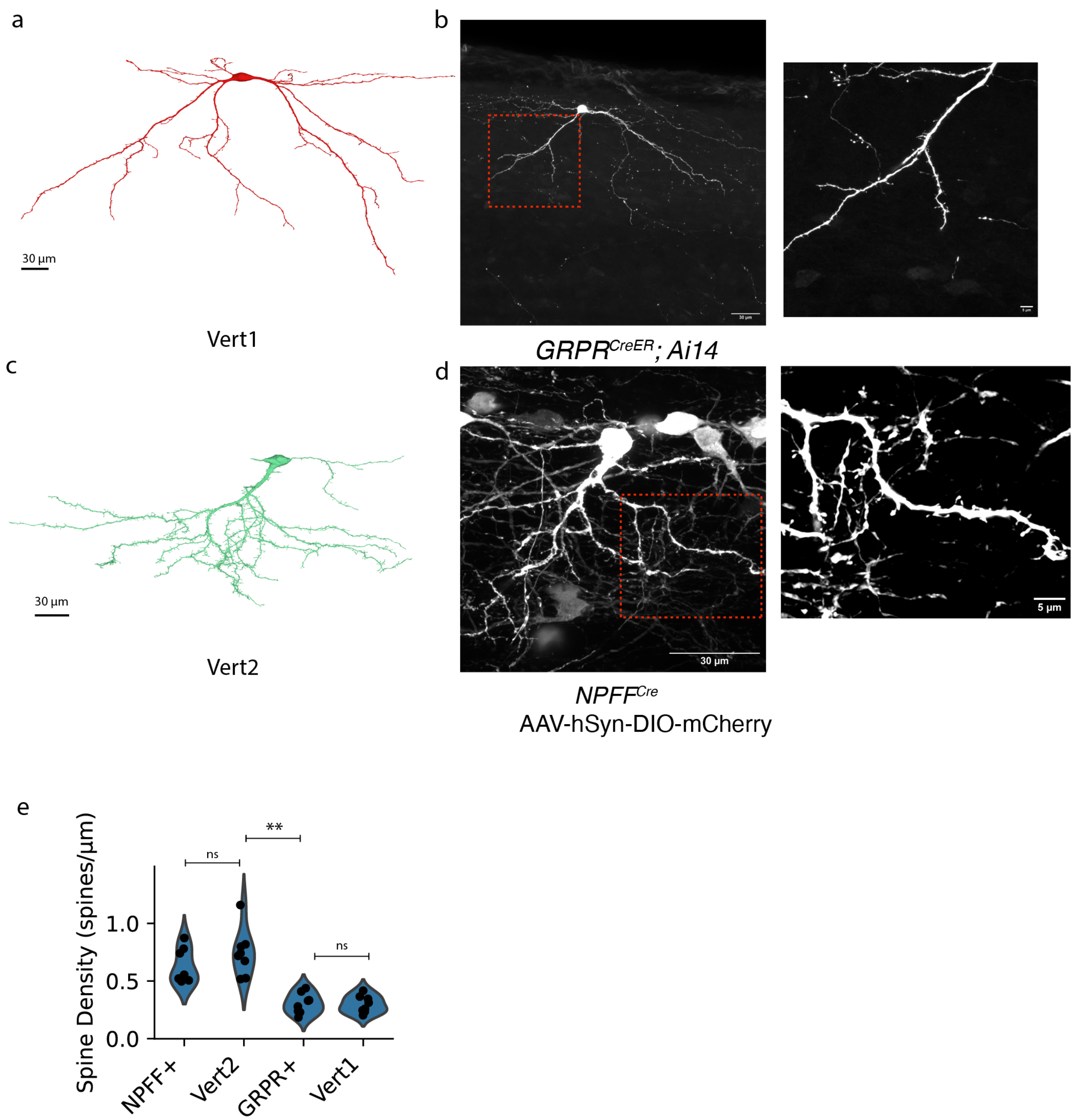


**Figure S6 | Genetically labelled Grpr⁺ and NPFF⁺ vertical cells visualized by light microscopy match with Vert1 and Vert2 neuron morphologies in the EM dataset.**

**a**, Example Vert1 neuron morphology reconstructed in the EM volume.

**b**, A sparsely labeled GRPR⁺ vertical cell from a *Grpr$^{CreER}$*; *Ai14* mouse. Right, higher-

bioRxiv preprint doi: https://doi.org/10.64898/2026.09.27.754843; this version posted October 1, 2026. The copyright holder for this preprint (which was not certified by peer review) is the author/funder, who has granted bioRxiv a license to display the preprint in perpetuity. It is made available under a CC-BY 4.0 International license.

magnification view highlights sparse dendritic spines.

**c**, Example Vert2 neuron morphology reconstructed in the EM volume.

**d**, A sparsely labeled NPFF$^+$ vertical cell (from an $NPFF^{Cre}$; AAV-hSyn-DIO-mCherry labeled mouse). Right, higher-magnification view highlights abundant dendritic spines.

**e**, Dendritic spine density comparison across light microscopy–labeled neurons and EM-reconstructed vertical cells. NPFF$^+$ vertical cells exhibit spine densities similar to Vert2 neurons and significantly higher than GRPR$^+$ vertical cells and Vert1 neurons. ($p = 2.91 \times 10^{-5}$; NPFF$^+$ vs Vert2, $p = 1$; Vert2 vs GRPR$^+$, $p = 1.1 \times 10^{-3}$; GRPR$^+$ vs Vert1, $p = 1$; NPFF$^+$ vs GRPR$^+$, $p = 1.2 \times 10^{-2}$, using a Kruskal–Wallis test, and a Dunn's posthoc with Bonferroni correction. NPFF$^+$ cell, n=7; GRPR$^+$ cell, n=8; V1 cell, n=8; V2 cell, n=8)

bioRxiv preprint doi: https://doi.org/10.64898/2026.09.27.754843; this version posted October 1, 2026. The copyright holder for this preprint (which was not certified by peer review) is the author/funder, who has granted bioRxiv a license to display the preprint in perpetuity. It is made available under a CC-BY 4.0 International license.

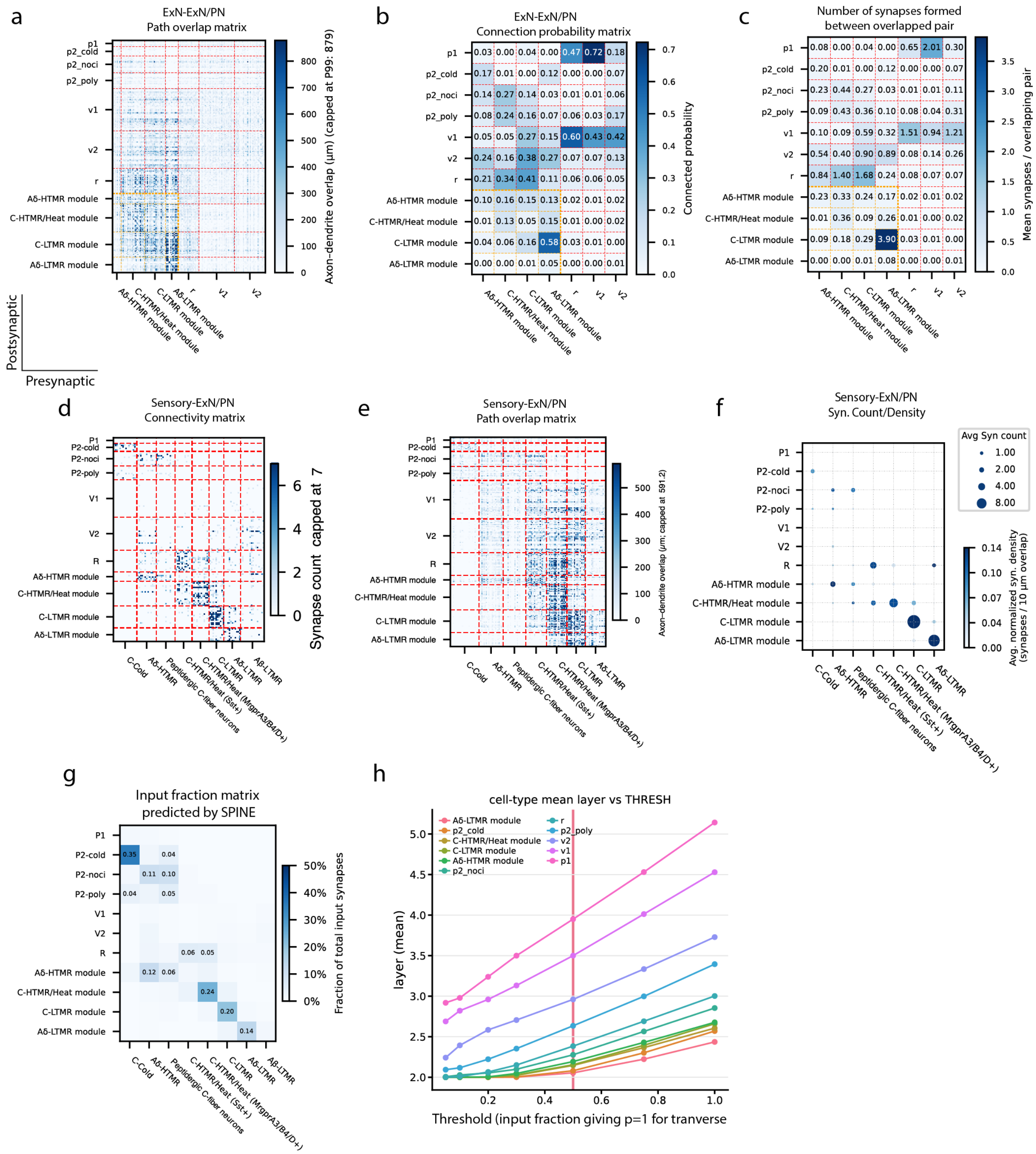


## Figure S7 | Connectivity and axo-dendritic overlap matrices between sensory neurons, ExN and PN subtypes

**a,** Matrix of axon–dendrite overlap for each ExN–PN pair, quantified as the total presynaptic axon cable length within 5 μm of the postsynaptic dendrite. The sensory module-to-sensory module region is delineated by yellow lines.

bioRxiv preprint doi: https://doi.org/10.64898/2026.09.27.754843; this version posted October 1, 2026. The copyright holder for this preprint (which was not certified by peer review) is the author/funder, who has granted bioRxiv a license to display the preprint in perpetuity. It is made available under a CC-BY 4.0 International license.

**b,** Matrix of connection probability, defined as the fraction of ExN–PN pairs with axon–dendrite overlap that formed one or more synapses. The sensory module-to-sensory module region is delineated by yellow lines.

**c,** Matrix of mean synapses count per overlapped neuron pair. The sensory module-to-sensory module region is delineated by the yellow lines.

**d,** Connectivity matrix summarizing synaptic connections among reconstructed sensory neurons, ExNs, and PNs.

**e,** Matrix of axon–dendrite overlap between reconstructed sensory neurons and postsynaptic spinal cord neurons, quantified as in **d**.

**f,** Mean synapse number and synapse density for sensory neuron-ExN/PN pairs with axon–dendrite overlap. Synapse density is calculated as the number of synapses per unit overlap length.

**g,** Fraction of total synaptic input contributed by each sensory subtype to reconstructed postsynaptic neurons estimated by SPINE. Only connections contributing, on average, more than 3.5% of the total synaptic input to the target ExN/PN subtypes are shown.

**h**, Robustness of traversal layers to threshold choice. Mean traversal layer of each ExN and PN subtype across the tested thresholds (2,000 iterations per threshold). Threshold choice altered the absolute layer values but not the relative ordering of ExN and PN subtypes.

bioRxiv preprint doi: https://doi.org/10.64898/2026.09.27.754843; this version posted October 1, 2026. The copyright holder for this preprint (which was not certified by peer review) is the author/funder, who has granted bioRxiv a license to display the preprint in perpetuity. It is made available under a CC-BY 4.0 International license.

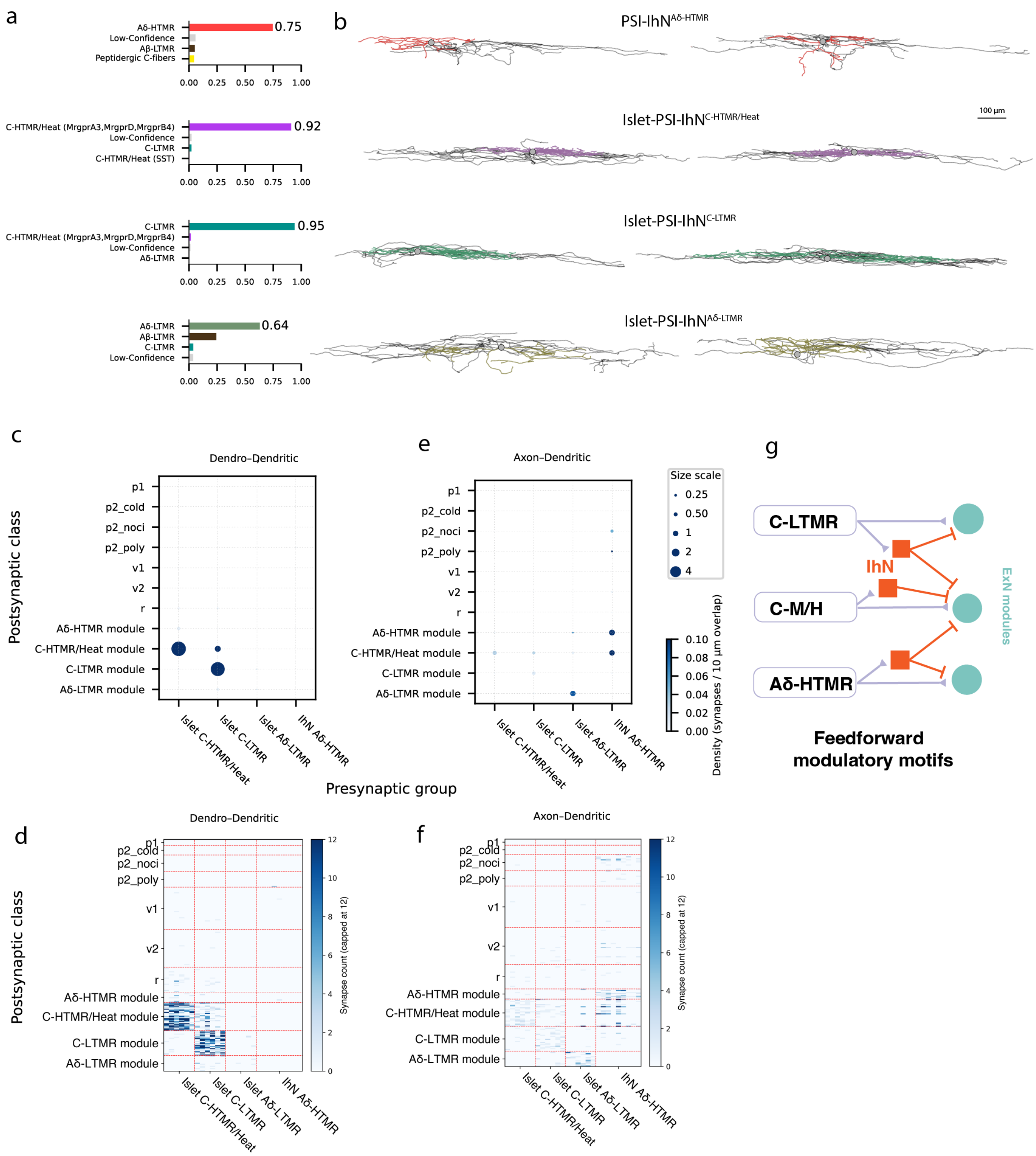


# Figure S8 | Connectivity between IhNs and ExNs

**a**, Sensory input composition of PSI-IhN$^{A\delta\text{-HTMR}}$, islet-PSI-IhN$^{C\text{-HTMR/Heat}}$, islet-PSI-IhN$^{C\text{-LTMR}}$, and islet-PSI-IhN$^{A\delta\text{-LTMR}}$ inhibitory populations. Each IhN subtype is named by its dominant sensory input.

**b**, Representative skeleton reconstructions of IhN$^{A\delta\text{-HTMR}}$, islet$^{C\text{-HTMR/Heat}}$, islet$^{C\text{-LTMR}}$, and islet$^{A\delta\text{-}}$

bioRxiv preprint doi: https://doi.org/10.64898/2026.09.27.754843; this version posted October 1, 2026. The copyright holder for this preprint (which was not certified by peer review) is the author/funder, who has granted bioRxiv a license to display the preprint in perpetuity. It is made available under a CC-BY 4.0 International license.

$^{\mathrm{LTMR}}$ neurons. Axons are shown in black.

**c**, Summary of dendro-dendritic synapses from inhibitory to excitatory subtypes. Dot size indicates mean synapse count for each subtype pair; color indicates mean synapse density per unit overlap (capped at the 95th percentile).

**d**, IhN inputs onto the ExN and PN subpopulations (dendro-dendritic synapses only). Islet-PSI-IhN$^{\mathrm{C\text{-}HTMR/Heat}}$ and Islet-PSI-IhN$^{\mathrm{C\text{-}LTMR}}$ uniquely form dendro-dendritic synapse.

**e,** Same as c, but for axo-dendritic synapses.

**f,** Same as d, but for axo-dendritic synapses.

**g**, Schematic illustrating heterotypic feedforward inhibition in the dorsal horn. Both C-LTMRs and Aδ-HTMR evoke feedforward inhibition to C-HTMR/Heat modules.

bioRxiv preprint doi: https://doi.org/10.64898/2026.09.27.754843; this version posted October 1, 2026. The copyright holder for this preprint (which was not certified by peer review) is the author/funder, who has granted bioRxiv a license to display the preprint in perpetuity. It is made available under a CC-BY 4.0 International license.

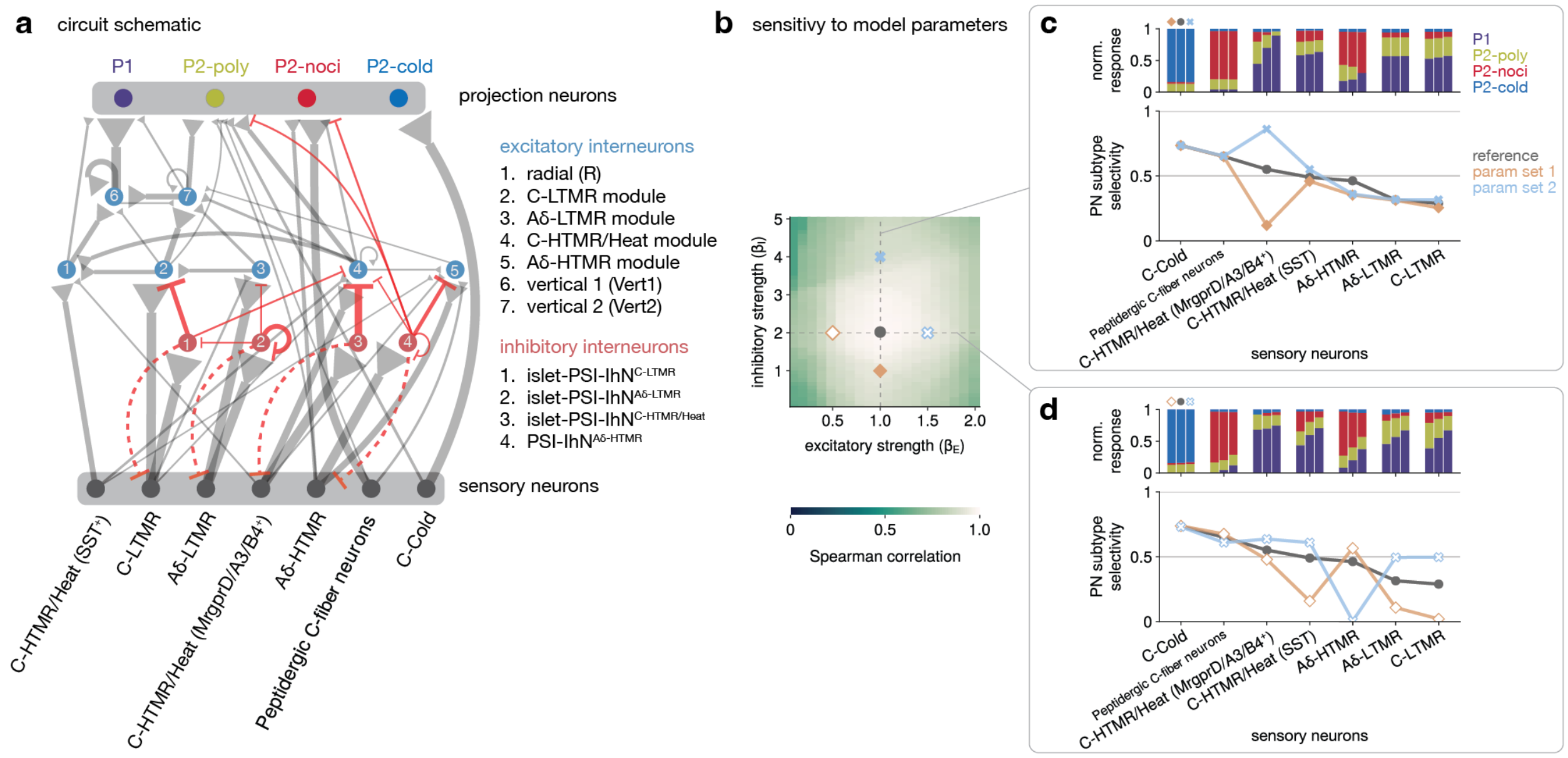


# Figure S9 | Wiring diagram of the model and sensitivity to model parameters.

**a**, Schematic of dorsal horn circuit derived from reconstructed sensory afferents and dorsal horn neurons. Each node represents a distinct neuronal population: sensory afferents (gray), excitatory interneurons (ExNs, blue), inhibitory interneurons (IhNs, red), and projection neurons (PNs, colored). Edge thickness between nodes is proportional to the corresponding weight in the connectivity matrices of **Figure 5a**, with weights capped at 0.5 and edges with weights < 0.065 omitted from the schematic for clarity; thus, the thickest edges correspond to a weight of 0.5 and the thinnest to 0.065. These weights reflect the relative contribution of each presynaptic population to the synaptic input of a typical postsynaptic neuron (of a given type) in the model (see Methods) and should be interpreted differently from the wiring diagram in **Figure 4**, where arrow thickness represents average synapse counts. Axoaxonic synapses mediating presynaptic inhibition are depicted as dashed lines, with thickness not scaled to synapse count, and are excluded from the model for simplicity. Note the strength of feedforward inhibitory motifs between sensory afferents, islet neurons and their corresponding ExN modules. Also note the strong direct pathways between C-Cold and peptidergic C-fiber neurons and their targets in the output (PN) layer.

**b**, Sensitivity of the model to parameters, specifically the strengths of excitation ($\beta_E$) and inhibition ($\beta_I$). $\beta_E$ scales the excitatory weights originating from ExNs, and $\beta_I$ scales the

bioRxiv preprint doi: https://doi.org/10.64898/2026.09.27.754843; this version posted October 1, 2026. The copyright holder for this preprint (which was not certified by peer review) is the author/funder, who has granted bioRxiv a license to display the preprint in perpetuity. It is made available under a CC-BY 4.0 International license.

inhibitory weights (see Methods). The heatmap shows the Spearman correlation between sensory-evoked normalized PN responses at the reference parameter set ($\beta_E = 1, \beta_I = 2$; gray circle) and those obtained for different ($\beta_E, \beta_I$) pairs.

**c**, Analogous to **Figure 5c**, but with three values of $\beta_I$: filled diamond, circles (reference), and filled cross, with $\beta_E$ fixed at the reference value. Normalized PN responses for each parameter set are shown as stacked bars (the center stacked bar corresponds to the reference parameter set). The corresponding selectivity metric is shown in the lower panel. Note that the identity of the preferred PN subtype is preserved across a wide range of parameter choices, despite changes in the degree of selectivity. The sensitivity of PN responses to $\beta_I$ differs across sensory stimulation conditions (see Methods).

**d**, Same as in **c**, except three values of $\beta_E$, with $\beta_I$ fixed at the reference value.
